# Harbor Seals (*Phoca vitulina*) Can Perceive Optic Flow under Water

**DOI:** 10.1371/journal.pone.0103555

**Published:** 2014-07-24

**Authors:** Nele Gläser, Björn Mauck, Farid I. Kandil, Markus Lappe, Guido Dehnhardt, Frederike D. Hanke

**Affiliations:** 1 University of Rostock, Institute for Biosciences, Sensory and Cognitive Ecology, Rostock, Germany; 2 University of Bochum, General Zoology and Neurobiology, Bochum, Germany; 3 University of Münster, Institute for Psychology, Münster, Germany; Institut Pluridisciplinaire Hubert Curien, France

## Abstract

Optic flow, the pattern of apparent motion elicited on the retina during movement, has been demonstrated to be widely used by animals living in the aerial habitat, whereas underwater optic flow has not been intensively studied so far. However optic flow would also provide aquatic animals with valuable information about their own movement relative to the environment; even under conditions in which vision is generally thought to be drastically impaired, e. g. in turbid waters. Here, we tested underwater optic flow perception for the first time in a semi-aquatic mammal, the harbor seal, by simulating a forward movement on a straight path through a cloud of dots on an underwater projection. The translatory motion pattern expanded radially out of a singular point along the direction of heading, the focus of expansion. We assessed the seal's accuracy in determining the simulated heading in a task, in which the seal had to judge whether a cross superimposed on the flow field was deviating from or congruent with the actual focus of expansion. The seal perceived optic flow and determined deviations from the simulated heading with a threshold of 0.6 deg of visual angle. Optic flow is thus a source of information seals, fish and most likely aquatic species in general may rely on for e. g. controlling locomotion and orientation under water. This leads to the notion that optic flow seems to be a tool universally used by any moving organism possessing eyes.

## Introduction

Movement is a fundamental characteristic of most organisms, and as soon as an organism, that is equipped with well-developed eyes, starts moving a visual motion pattern is elicited on the retina known as optic flow [1], [2]. Naturally, optic flow has been shown to be a powerful tool in a wide range of species to be used for goal directed locomotion, controlling velocity of movement, centring or collision avoidance. Optic flow information is detected by species irrespective of e. g. type of eye, including compound eyes as well as single-chambered eyes, their visual environment, including even a nocturnal insect operating in dim light conditions [3], or their mode of locomotion, including flying, walking, and climbing organisms (see e. g. [4]–[10]).

However so far, the analysis of optic flow has mainly focused on species inhabiting the aerial environment. Underwater optic flow has, until now, only attracted attention in investigations examining the fish optomotor response (see e. g. [11]–[16]), also in the context of fish rheotropism [17], [18], and a recently published study has aimed at investigating fish optic flow use in an optic flow tunnel [19]. Generally, a plethora of optic flow sources are available in the underwater environment. Aquatic species could gain optic flow information, when moving over the sea bottom or below the water surface. Additionally, they experience optic flow from nearby particles, when moving through particulate matter. The latter is particularly remarkable, as particles drastically reduce visual resolution [20], [21] and as their presence, along with sparse illumination and the lack of reliable landmarks at most locations, has so far been considered to argue against visual orientation in aquatic species. Thus, if optic flow was perceived by aquatic species, as previous fish studies suggest [11]–[19], it would provide valuable information about self-motion relative to the environment even or particularly under conditions, in which visual orientation seemed limited.

In this study, we examined underwater optic flow perception for the first time in a semi-aquatic mammal, the harbor seal. Harbor seals could benefit from optic flow information offshore where the amount of external information might often be drastically reduced and where visual information is reduced to optic flow information elicited e. g. by movement through particulate matter. In a first approach to this topic, we simulated a forward movement (pure translation) through a cloud of dots, mimicking a movement through particles, on an underwater projection. The seal was able to perceive optic flow and showed high accuracy in detecting deviations from the simulated heading, which, in linear movements, corresponds to the focus of expansion (FOE), the singular point from which all visual motion seems to emanate.

## Material and Methods

### Ethics statement

The experiments were carried out in accordance with the European Communities Council Directive of 24 November 1986 (86/609/EEC). According to § 8 of the German Animal Welfare Act of 18 may 2006 (BGB I. I S. 1206, 1313), experiments conducted in this study were not subject to approval or notification, since they did not cause pain, suffering or injuries to the animals.

### Experimental animal

Experiments were conducted with an experimentally experienced [22]–[26], eight-year old male harbor seal that was born in captivity at Zoo Duisburg, Germany. During the time of the experiments, the harbor seal was housed together with seven other harbor seals in two adjacent and connected freshwater pools (300 m^3^, and 600 m^3^) with a maximum water depth of approximately 2 m at the Marine Science Center at Zoo Cologne, Germany. Water quality was maintained by a continuous inflow of freshwater and regular water changes. Both pools offered the seals many haul-out places. Besides experimental sessions, all seals participated in several daily training sessions, including e. g. health checks and husbandry training. The Marine Science Center was regularly visited by the zoo's veterinarians. Testing took place up to twice a day for typically five days per week. The main part of the daily food amount of 2–5 kg freshly thawed herring was fed during the experiments as primary reinforcement. Additional food was given during the numerous daily training sessions.

### Optic flow stimuli

**Figure 1:**
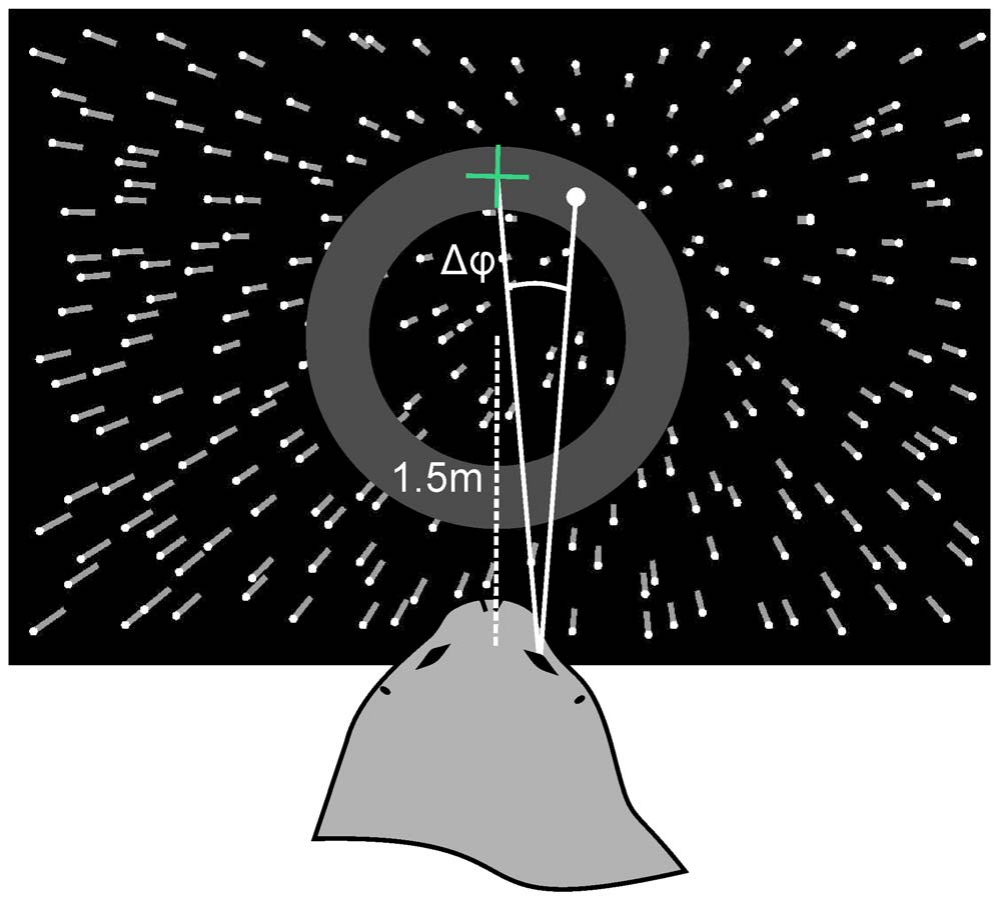
Optic flow projection. A three dimensional cloud of dots was presented to the harbor seal on an underwater projection screen. The FOE of the optic flow field (white dot, dot not shown during experiments) could occur on 28 positions all covered by a ring-shaped mask (grey circle). A cross was superimposed on the optic flow field at a pre-programmed angular distance (delta phi) to the FOE and could either match (“no go”-trial) or deviate from the FOE (“go”-trial, illustrated in this figure).

Optic flow stimuli (Figure 1) were presented simulating a forward self-motion of the seal on a straight path (pure translation). The stimuli were produced by randomly positioning 500 white dots in an otherwise empty virtual three dimensional space, that appeared black on the underwater projection screen, and moving the camera linearly through the scene with a speed of 2 m/s, a typical swimming speed of harbor seals (see e. g. [27]). Thus, speed of the dots increased with eccentricity and decreased with distance in depth identical to the speeds experiences during real motion through an underwater volume. Dots remained visible until leaving the projection but did neither grow in size nor increase in brightness as they came nearer. For the observer this resulted in an animated picture, in which all dots seem to emerge from the focus of expansion (FOE) and travel straight to the periphery. The FOE was programmed to be in one out of 28 positions (visual angle phi) with 7 positions in each of the four quadrants of the projection and eight areas in which the FOE could occur. In order to prevent the seal from using local cues at the position of the FOE, such as an increased density of points at the FOE, an 8 deg broad ring-shaped mask ranging from a radius of 22 deg to 29.5 deg masked all possible positions of the FOE and their direct vicinity.

### Experimental setup and procedure

**Figure 2:**
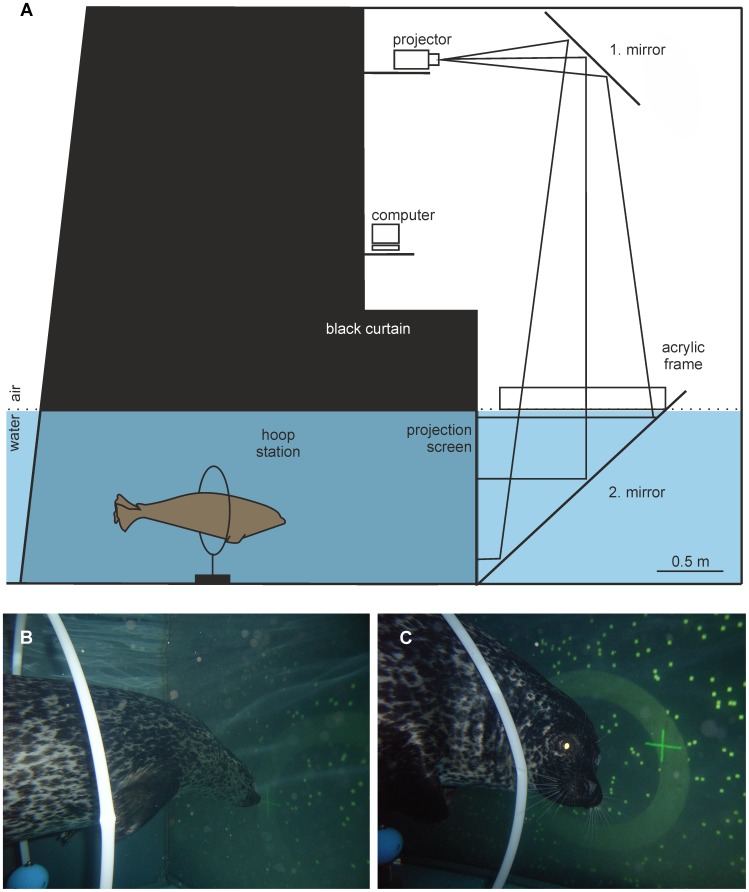
Experimental setup for the determination of the threshold of heading perception from virtual optic flow and response behavior of the harbor seal. A The computer generated optic flow field was back-projected by a projector and via a mirror system onto an underwater projection screen installed inside a projection chamber. For a high-quality underwater projection, the water surface was calmed by an acrylic frame, and the experimental area in front of the projection chamber was shaded by a black curtain. Inside this area, the animal was stationing underwater in a hoop station with the eyes at 1.5 m distance to the projection. During trials, the experimenter (not displayed) stayed outside the experimental area in order to avoid giving secondary cues. Scale 0.5 m. B In a “no go”-trial, in which the superimposed cross matched the FOE, the animal had to touch the cross with its snout in order to be rewarded. C In a “go”-trial (compare with Figure 2), in which the cross deviated by a pre-programmed angular distance from the FOE, the seal had to turn away from the projection screen and touch the hoop station with its snout.

Stimuli were back-projected by an Epson EMP-9100 projector onto a 2×3 m underwater projection screen (Figure 2A). The complete area in front of the projection was covered with a black curtain ensuring a good projection and constant experimental conditions. The experimenter stayed outside of the experimental area in order to avoid secondary cueing and started a trial by switching on the projection. On command, the seal entered the experimental area and positioned itself in a 0.8 m diameter stationing hoop with the eyes at 1.5 m distance to the projection. Viewed from the stationing hoop, the projection filled a visual field of 73.7 deg ×47.2 deg. After the animal had stationed for five seconds, a white cross was superimposed on the optic flow field. The cross could either match or deviate from the FOE. If the cross matched the FOE, a correct response was scored if the animal touched the cross on the projection with its snout (“no go”-trial, Figure 2B). If the cross deviated by an angular distance (delta phi) from the actually presented FOE, a correct response was scored if the animal turned away from the projection screen and touched the stationing hoop (“go”-trial, Figure 1 and Figure 2). Each correct response was rewarded with a piece of fish, an incorrect response was followed by a verbal “no”. After each experimental trial, the projection was switched off, and the animal was called out of the experimental area.

In order to obtain a threshold of heading perception when simulating a translatory movement, defined as the angular distance between cross and FOE that could be detected in 50% of the deviation trials, the angular distance between FOE and cross was varied among trials. Each angular distance could be presented at any position of the FOE. Altogether 289 combinations of FOE and cross were shown to the seal. All angular distances between the cross and the FOE were presented 53 to 78 times following the method of constant stimuli [28]. An estimate of the threshold of heading perception was obtained during pretraining enabling the deployment of four angular distances between cross and FOE presumably above, 1.3 deg, 2.7 deg, 5.3 deg, and 10.6 deg, and two angular distances presumably at/below the estimated threshold, 0.3 deg, and 0.7 deg, for data collection. The seal was presented with more angular distances between FOE and cross above than below threshold in order to sustain a high motivation level. Each position of the FOE was shown 19 to 30 times overall balancing the frequency with which the various FOE positions within the quadrants were displayed to the seal. Experimental sessions consisted of 40 to 50 trials with equal likelihood of “go”- and “no go”-trials. In these sessions, the various FOE positions as well as the various angular distances between cross and FOE were presented in random order.

### Data analysis

We fitted the data using an exponential function and interpolated the 50% threshold representing the visual resolution of the seal for optic flow. Significance was calculated in IBM SPSS Statistics Version 20 using a χ^2^-Test against chance performance.

## Results

**Figure 3:**
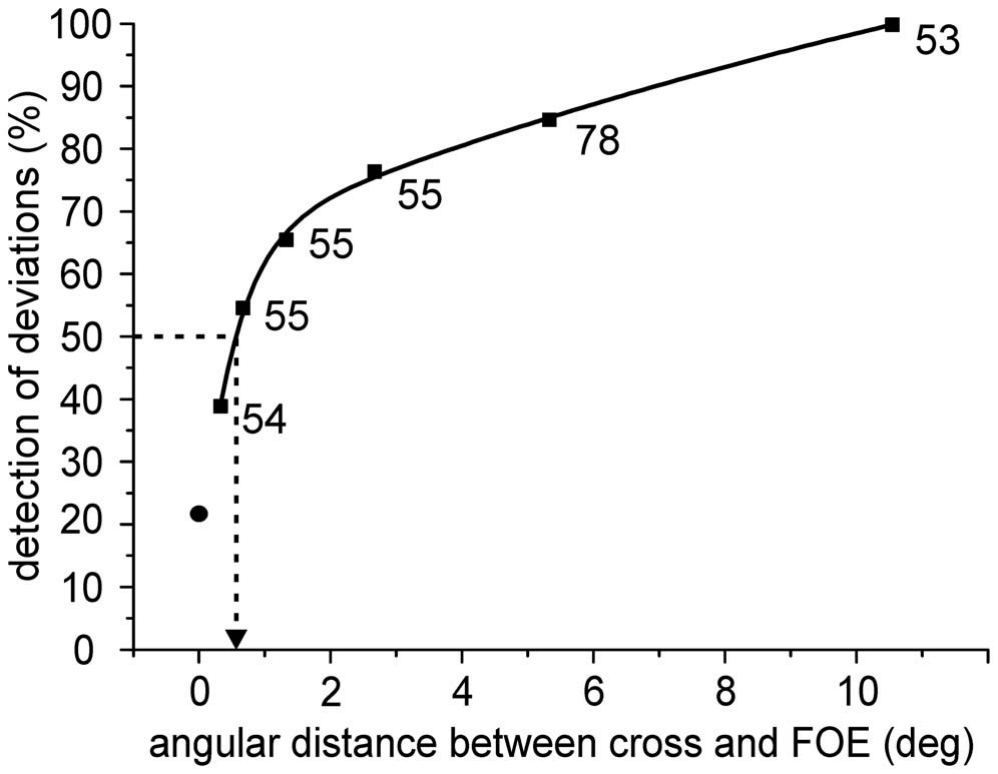
Accuracy of heading perception of a harbor seal. The harbor seal's ability to detect deviations of the cross from the FOE (in %) is plotted as a function of the angular distance between FOE and cross (in deg). The threshold of heading perception, defined as the angular distance between FOE and cross (delta phi) that could be detected with a performance of 50%, was interpolated by fitting an exponential function to the data (black curve; r^2^ = 0.99). The resulting threshold of 0.6 deg is indicated by a black arrow pointing to the x-axis. Numbers at the data points indicate the number of trials per angular distance. The seal's false alarm rate of 21.7% (N = 351) is indicated at an angular distance of 0 deg (closed circle).

The harbor seal was able to accurately perceive the location of the FOE, the direction of heading of the simulated translation. The ability of the seal to detect deviations of the cross from the FOE as a function of angular distance is shown in Figure 3. The seal's 50% threshold was at an angular distance of 0.6 deg. During the trials, in which the cross matched the FOE, the seal showed a low spontaneous response rate (false alarms) of 21.7% corresponding to a high performance of 78.3% correct choices (p<0.01).

The experimental animal accomplished the task almost effortlessly. When the seal was allowed to enter the experimental area, it directly aligned its body with the flow field, in particular it aligned with the specific FOE that could occur on 28 positions concentrated in eight areas, as if it was intending to swim with the flow indicating that the two-dimensional flow field created an impression of self-motion in the seal. Furthermore the seal gave its response within a second after the cross was superimposed on the simulation.

## Discussion

Our seal showed a performance that is congruent with heading perception from optic flow which is the prerequisite to use optic flow for self-motion control, which, however, awaits further examination. Optic flow seems to be a clear and powerful cue for harbor seals as indicated by the low threshold of heading perception and the ease, with which the seal was operating in our optic flow experiment including the fast reaction time and acquisition of the basic task, which was faster than e. g. in any other visual experiment conducted with harbor seals before. Right from the beginning, the seal was going with the flow which is an indication that it experienced an illusion of self-motion in our virtual environment. This might suggest that the virtual environment of a three dimensional dot cloud closely resembles the scenario a harbor seal encounters when moving through a cloud of particles and that the projection does not represent a drastic simplification of the natural environment. With the documented sensitivity to optic flow harbor seals can most likely exploit optic flow information from all kind of sources, originating also from movement close to the sea floor or the water surface. More generally, based on the results obtained with harbor seals and fish, optic flow might be a universal tool to be used by moving organisms in the aerial as well as the underwater environment as has already been pointed out by Gibson [2].

However, the underwater optic flow condition differs in two important aspects from the condition encountered by terrestrial or flying animals. First, the visual depth range is most often restricted to optic flow from nearby regions only, which is known to cause computational problems in distinguishing translational from rotational self-motion components [29], [30]. And second, concerning optic flow induced by particles, water movements are likely to violate the requirements of environmental rigidity, which underlies all current computational algorithms for the recovery of self-motion parameters from optic flow [31], [32]. If aquatic animals thus use underwater optic flow, they may have developed novel strategies for its analysis.

The very precise performance of our seal compares favorably with the accuracy of heading perception previously obtained in psychophysical experiments in humans (e. g. [33], [34]) and monkeys [8]–[10]. Given that our harbor seal interprets optic flow displays with a similar precision as e. g. humans suggests that seals might be able to use optic flow in contexts, in which humans rely on this source of visual information such as goal directed locomotion [1], [35], [36]. Considering a typical swimming velocity of harbor seals of 2 m/s (e. g. [27]), the precision the seal exhibited would be sufficient for the usage of heading judged from optic flow for goal directed locomotion [36]. Harbor seals might also benefit from optic flow information for collision avoidance [37], and might estimate further parameters from optic flow such as travelled distance (e. g. [38]), one prerequisite for path integration. Path integration based on optic flow cues, or on any other cue, would be a very useful tool for underwater orientation, as it allows to integrate a vector, which would guide the animals e. g. back to their haul-out sites. Harbor seals could benefit from path integration even under conditions in which aquatic organisms cannot rely on other mechanisms of visual orientation such as the sun, stars or landmarks.

Our finding of underwater optic flow perception in harbor seals supports the role of vision for the control of locomotion and orientation in marine mammals, which has been discussed controversially in the past [39]–[43]. Underwater visual orientation is thought to be limited e. g. in harbor seals as harbor seals probably cannot use polarized light [44], landmarks are most likely sparse underwater, low light levels or darkness prevail underwater, and even in shallow waters and under good light conditions dissolved and particulate matter in the water column often cause turbidity that impedes sharp vision and object detection [20], [21]. However, in this environment, optic flow information is richly available irrespective of the animal's position within the water column. Consequently, the seal's ecology might have encouraged the perception and most likely the use of optic flow information to allow for underwater visual orientation and self-motion control.
